# Removal of the Uterus for "Unavoidable Hæmorrhage"

**Published:** 1899-02-18

**Authors:** 


### REMOVAL OF THE UTERUS FOR "UNAVOIDABLE HÆMORRHAGE."

Mr. Lawson Tait[1] suggests removal of the uterus by the proceeding known as the "Tait-Porro" operation in certain cases of unavoidable hæmorrhage. He quotes Simpson to the effect that "all obstetric authors seem to agree on this point, that there is no one complication in midwifery attended with more anxiety to the practitioner, and few, if any, with more real danger to the patient than cases of unavoidable hæmorrhage from presentation of the placenta." He feels sure that the full mortality from the condition is not yet known, so many patients die from secondary complications which are not fatal till many days after delivery, the deaths being thus returned, not as cases of placenta prævia, but as peritonitis, &c. Moreover, in deciding upon what to do in such cases one is met by the conflicting claims of the mother and the child, for it must frequently be the case that what is safest for the one is likely to involve the death of the other, in regard to which one has to bear in mind that the Church of Rome rules that in such cases the living birth of the child is all important, and that when the interests of the mother and child conflict, those of the former must yield. Under such circumstances the question is whether one may not, by removing the uterus, save both the mother and the child. The following case, which Mr. Lawson Tait had with Dr. Herbert Simpson, is given in support of this contention: "The patient was young and slightly built, and had as bad a history of hæmorrhage as a woman well could have. She had always had 'something wrong' inside, and had been a regular consumer of ergot for years. She had been twice curetted and cauterised. She had nearly lost her life from post-partum hæmorrhage in her second confinement, had had several miscarriages with severe losses, and I found her in her fourth confinement at full time with cervix closed and rigid, the liquor amnii drained off, the uterus firmly contracted over the child, and yet she had been bleeding with alarming profusion for five hours, in spite of many other and orthodox points of treatment which Dr. Simpson had employed. The child was easily ascertained to be alive. . . . I confess that the recollection of previous experiences made me hesitate to do what was clearly necessary, forcibly dilate the cervix and extract the placenta, whether I followed that by version or not, whether or not I left the after-labour to nature and the child to its fate. An alternative occurred to me, to which I was prompted by the splendid success which has followed it in my hand and in those of others under different yet very analogous circumstances—removal of the uterus. . . . I therefore proposed hysterectomy, and . . . performed it . . . The patient made a straightforward recovery, interrupted only by occasional rectal distension. A fine female child was born alive." The operation performed may be thus briefly described: After a four-inch incision is made as usual for abdominal section, a loop of drainage tube is carried over the uterus and down to the pelvic sulcus. This is tightened sufficiently to strangle the circulation and then tied. The uterus is then opened, and the child and placenta are delivered. The uterus is then drawn out of the abdomen, a stout needle is passed through the cervix immediately above the ligature, the body of the uterus is cut away, the stump is fixed in the corner of the wound, the latter is accurately closed after the necessary cleansing, and the stump is dressed with chloride of iron or other dessicant.

[1] Lancet, Feb. 11.

